# The effects of the menstrual cycle on physical and psychological parameters in female athletes

**DOI:** 10.1038/s41598-026-47706-0

**Published:** 2026-04-09

**Authors:** Marc Niering, Valentina Schilling, Rainer Beurskens, Johanna Seifert

**Affiliations:** 1 Department of Psychiatry, Social Psychiatry, and Psychotherapy, Hannover Medical School, Carl-Neuberg Straße 1, 30625 Hannover, Germany; 2 School of Sports, Psychology and Education, Triagon Academy Munich, Ismaning, Germany; 3 Department of Health and Social Affairs, FHM Bielefeld - University of Applied Sciences, Bielefeld, Germany; 4 Institute of Biomechanics and Neurosciences, Nordic Science, Hannover, Germany

**Keywords:** Health care, Physiology, Psychology, Psychology

## Abstract

Hormonal fluctuations across the menstrual cycle may influence physical performance and psychological well-being in female athletes. However, current evidence remains heterogeneous, partly due to inconsistent cycle phase determination and task-dependent outcome measures. This study aimed to describe phase-dependent changes in physical and psychological parameters across six hormonally validated menstrual cycle phases and to examine associations between these domains. Eighteen eumenorrheic female athletes from various sports (age 18–30 years; 23.6 ± 3.8 years) were assessed across six menstrual cycle phases. Dynamic maximal strength in the half squat, isometric handgrip strength, and psychological variables including POMS subscales, motivation, sleep quality, and perceived exertion were measured. Significant phase effects were observed for half-squat performance, isometric handgrip strength, and several POMS subscales. Main effect *p* values ranged from *p* = .029 to *p* < .001, with partial eta-squared effect sizes between *η*²*p* = .13 and 0.98. Half-squat performance peaked in the late follicular phase and at ovulation and was lowest in the late luteal phase. Handgrip strength reached its highest values during the late luteal phase. Psychological measures showed increased fatigue and depression scores and reduced vigor in the late luteal phase. No phase effects were found for motivation, sleep quality, or perceived exertion (*p* = .424–0.698). At the individual level, half-squat performance was negatively associated with POMS depression scores (*r* = − .60, *p* = .009). All other correlations were non-significant, with *r* values ranging from − 0.44 to − 0.47 and *p* values between 0.052 and 0.849. A pattern-oriented and individually adaptive training approach is recommended, including the development of menstrual cycle literacy among athletes and support staff. A monitoring strategy using a small number of informative strength and well-being markers, combined with regular feedback, may help adjust training loads to individual menstrual cycle patterns.

## Introduction

Scientific interest in cycle-sensitive training prescription has increased markedly in recent years, as hormonal fluctuations across the menstrual cycle are considered relevant modulators of physical performance and recovery. The menstrual cycle is regulated by the interaction of the hypothalamus, pituitary gland, and ovaries and typically spans approximately 26 to 32 days. Following the framework described by McNulty et al.^1^, the cycle can be divided into the early follicular (EF) and late follicular (LF) phases, ovulation (OV), and the early (EL), mid (ML), and late luteal (LL) phases.

At the onset of the cycle (EF), circulating estrogen and progesterone concentrations are low. Estrogen levels rise toward ovulation, whereas progesterone predominates during the luteal phase before both hormones decline toward the end of the cycle. These hormonal fluctuations have been shown to influence energy metabolism, thermoregulation, neuromuscular activation, and psychological well-being^1,2^. More recent evidence suggests that endocrine variations may also modulate metabolic processes, inflammatory regulation, and recovery, highlighting the need to jointly consider physiological and psychological parameters when examining menstrual cycle effects^3^.

Despite growing research activity, the overall evidence remains heterogeneous. Inconsistencies in cycle phase determination, timing of measurements, small sample sizes, and task-specific outcome measures limit comparability and practical transferability, as emphasized by several authors^2,4,5^. For strength performance, mean effects across cycle phases are generally small or absent, with observed differences often being task dependent^4^. Dynamic and isometric strength measures may differ in their sensitivity to hormonal fluctuations due to task-specific neuromuscular demands. Previous research suggests that menstrual cycle effects on strength performance are task-dependent and may vary depending on the specific performance modality^1,4^. Therefore, the inclusion of both dynamic and isometric measures in the present study aimed to capture potentially distinct patterns across different types of neuromuscular performance.

The recent meta-analysis by Niering et al.^6^, identified subtle, modality-specific patterns. Performance tended to be lower during the EF, whereas the LF was associated with higher values in dynamic and, in some cases, isometric maximal strength. Isokinetic outcomes showed advantages around ovulation. However, the authors highlighted substantial interindividual variability and limited sample sizes. Complementary findings indicate that neuromuscular fatigue may increase during the LF, potentially contributing to performance decrements^7^.

Cycle-dependent alterations in the testosterone-to-cortisol ratio may further influence readiness and adaptation, offering an additional explanation for heterogeneous findings^8^. Single studies with rigorous phase verification underscore the context dependency of performance outcomes. For example, Romero-Moraleda et al.^9^, reported no phase-related differences in half-squat performance without caffeine supplementation, whereas higher movement velocities were observed during the LF and OV following caffeine intake. Evidence regarding cycle-related injury risk remains similarly inconsistent, as variations in definitions, assessment windows, and validation strategies complicate cross-study comparisons and limit interpretability^10–12^.

In parallel, psychological parameters have received increasing attention. Mood-related changes across the menstrual cycle have been reported, with higher vitality in earlier phases and increased fatigue and depressed mood toward cycle end. These effects have been linked to hormonal influences on serotonergic and dopaminergic systems and their interaction with stress regulation^2^.

Regarding sleep, a relatively consistent pattern emerges at the perceptual level. Subjective sleep quality is more frequently rated as impaired during the EL, whereas objective sleep parameters do not consistently vary across phases. Current evidence highlights interactions between hormonal fluctuations, sleep duration, sleep architecture, and cardiovascular markers, supporting the use of individualized, symptom-based strategies^13,14^.

Experimental findings further suggest that recovery and well-being may fluctuate across the cycle, while motivation does not necessarily follow the same pattern. This dissociation supports the targeted inclusion of psychological markers in training regulation^15,16^.

Against this background, the present study aimed to examine whether dynamic and isometric maximal strength, as well as selected psychological variables, differ across six hormonally validated menstrual cycle phases in eumenorrheic female athletes. In addition, potential associations between physical and psychological parameters were explored to identify patterns relevant for training practice. It was hypothesized that dynamic and isometric strength as well as selected psychological parameters would vary across menstrual cycle phases, while associations between physical and psychological variables were expected to be small and inconsistent. By applying a consistent, hormone-validated study design, this work seeks to refine heterogeneous findings and contribute to the development of evidence-based, cycle-sensitive training strategies^1,17^ .

## Methods

### Participants

The study was conducted using a repeated-measures design within the same individuals to capture cycle-dependent changes at the intraindividual level while minimizing the influence of interindividual variability^18^. To determine the required sample size, an a priori power analysis was conducted using G*Power 3.1^19^. Assuming a medium effect size (*f* = 0.30), a significance level of α = 0.05, and a statistical power of 1 − β = 0.80, a minimum sample size of 15 participants was required. With 18 athletes included, the target sample size was exceeded.

A post hoc power analysis based on the observed effect sizes of the significant main effects (*η*²*p* =.21–0.98) indicated achieved power values ranging from 1 − β = 0.88 to > 0.99, confirming that the analyses were sufficiently powered to detect effects of at least medium magnitude^20,21^.

The sample comprised 18 physically active women aged 18 to 30 years (23.6 ± 3.8 years). All participants reported a regular, natural menstrual cycle and did not use hormonal contraception. Training volume was at least three sessions per week, indicating adequate training status and familiarity with resistance-based performance testing.

Participants were classified as “Tier 2” according to the Participant Classification Framework to ensure transparent reporting of training status and menstrual cycle characteristics and to enhance comparability with existing research^22^. Mean body mass was 60.9 ± 5.7 kg, and mean body height was 166.0 ± 4.6 cm.

To reflect a natural cross-section of physically active women, the sample was deliberately designed to be heterogeneous, and no restriction by sport discipline was applied. Triathlon and artistic gymnastics were each represented by three athletes (16.7% each), volleyball and strength sports by two athletes each (11.1%), and additional disciplines including soccer, handball, tennis, basketball, swimming, rowing, and judo by one athlete each (5.6% each). This distribution supports the external applicability of the findings beyond single sport disciplines.

In total, 108 measurements were included in the analysis, corresponding to six measurement time points per participant. The testing protocol and measurement order were identical across all cycle phases, ensuring that observed differences cannot be attributed to procedural or order effects.

### Determination of menstrual cycle phases

The assignment of measurement time points to menstrual cycle phases was based on LH-based phase verification using urinary luteinizing hormone (LH) ovulation tests. No additional hormonal measurements (e.g., estradiol or progesterone) were conducted; therefore, ovulation and luteal phase sufficiency could not be biochemically confirmed. The first clearly positive test result, indicating the LH surge, was used to define the periovulatory window. All remaining measurement time points were scheduled in relation to both the onset of menstruation and the detected LH surge.

This approach follows methodological recommendations from comprehensive reviews, which emphasize physiological anchoring compared with purely calendar-based methods in order to reduce misclassification and enhance internal validity^1^. These guidelines further emphasize that studies lacking hormonal confirmation should be downgraded in evidence quality, underscoring the importance of physiologically anchored phase determination for the interpretation of menstrual cycle effects^17^. Accordingly, the present study used the LH surge as a physiological anchor, with all measurement windows positioned relative to this event and the onset of menstruation.

### Data collection

The experimental design aimed to capture phase-related changes in psychological and physical parameters under constant conditions at the intraindividual level. Each participant completed assessments at six measurement time points corresponding to the hormonally validated menstrual cycle phases. At each session, a fixed testing sequence was applied to minimize order effects and ensure comparability across phases, as recommended for repeated-measures designs^23^.

Mood was assessed first using the validated short version of the Profile of Mood States (POMS-16). This version includes the subscales vigor, fatigue, depression, and anger and demonstrates a stable factor structure and high internal consistency. In the validation study by Petrowski et al.^24^, Cronbach’s *α* values ranged from 0.84 to 0.94, indicating very good reliability. The POMS-16 is therefore considered a reliable and valid instrument for assessing current affective states.

Motivation and sleep quality were assessed using visual analogue scales (VAS). The VAS is an economical tool for measuring subjective perceptions and shows high test–retest reliability (*r* =.80–0.94) as well as good construct and content validity^25^. Due to its sensitivity to short-term state changes, the VAS is particularly suitable for repeated assessments in cycle-based study designs^26^.

Maximal strength was assessed using a one-repetition maximum test in the half squat. This procedure demonstrates very high test–retest reliability, with an intraclass correlation coefficient *(*ICC*)* of 0.97 and a standard error of measurement of 1.9%, indicating high measurement precision^9^. The half squat is considered a valid measure of dynamic lower-body strength and is strongly associated with sport-specific performance parameters such as jumping and acceleration ability.

Isometric handgrip strength was used as an indicator of general neuromuscular performance. Handgrip strength is a reliable and valid biomarker with high reproducibility (ICC > 0.90) and low susceptibility to measurement error^27^. Recent reviews describe handgrip strength as a sensitive indicator of physical performance and muscular function that remains stable across repeated assessments^28^. Standardized testing procedures, including identical arm position and grip configuration, were applied to further ensure objectivity.

Subjective exertion was assessed using the Borg rating of perceived exertion scale, which shows strong correlations with physiological parameters such as heart rate and oxygen uptake (*r* =.80–0.90)^29^. The scale is considered reliable, valid, and sensitive to short-term changes in exercise intensity and is widely used in sport science research to quantify perceived effort.

To minimize methodological variability, several standardization procedures were implemented. All measurements were conducted using identical equipment settings, standardized instructions, and a brief, consistently structured familiarization trial. This trial served to stabilize task execution and scale usage while reducing initial uncertainty. The testing order remained identical across all cycle phases, ensuring that potential order or fatigue effects were held constant. All testing sessions were conducted in the afternoon. While testing times varied slightly between participants (approximately 2–3 h), they were kept consistent within each participant across all measurement time points. Participants were instructed to maintain their usual nutritional habits and to avoid strenuous exercise prior to testing sessions. Data were recorded using standardized collection forms and were checked for plausibility prior to statistical analysis.

These procedures align with established recommendations for repeated-measures designs, in which consistency of testing procedures and data handling is a key prerequisite for internal validity^23^. In addition, care was taken to ensure phase-consistent scheduling, such that the sequence of the six measurement time points followed the same structure for each participant. This approach further enhanced comparability both within individuals and across cycle phases.

### Statistical analysis

Statistical analyses were performed using jamovi (version 2.6.13). Graphical visualization of means and standard deviations across cycle phases was conducted using GraphPad Prism (version 10.2). Descriptive statistics were calculated for all variables at each menstrual cycle phase.

To examine cycle-dependent changes, separate repeated-measures analyses of variance (ANOVA) were conducted for each dependent variable. This approach accounts for within-subject dependency and interindividual variability^23^. Sphericity was assessed using Mauchly’s test. When violated, Greenhouse–Geisser–corrected results were reported; Huynh–Feldt corrections were applied in cases of minor deviations. Significant main effects were followed by Bonferroni-adjusted pairwise comparisons. Effect sizes were quantified using partial eta squared (*η*²p) and interpreted as small (0.01), medium (0.06), or large (0.14) effects^30^.

Associations between physical and psychological variables were examined using Pearson correlation analyses. To ensure independence of observations, values were averaged across the six phases for each participant prior to correlation analysis. Holm correction was applied to adjust for multiple testing.

Results of repeated-measures analyses are reported as *F* values with corresponding degrees of freedom (corrected where applicable), *p* values, and *η*²p. Statistical significance was set at *p* <.05. Correlation results are presented as Pearson’s *r* with 95% confidence intervals and Holm-adjusted *p* values.

## Results

### Overview of phase-related changes

Descriptive statistics for all physical and psychological variables across menstrual cycle phases are presented in Table 1. Ratings of perceived exertion, motivation, and sleep quality showed minimal variation without distinct phase-specific peaks.

In contrast, the POMS subscales demonstrated clearer cyclical patterns. Vigor was highest during the follicular phases and lowest in the late luteal phase, whereas fatigue increased progressively and reached its maximum in the late luteal phase. Depression scores were lowest around ovulation and during the early luteal phase and highest at the cycle end. Anger remained generally low across phases.

**Table 1:** Descriptive statistics (M ± SD) for physical and psychological variables across six menstrual cycle phases.

Variable	EF	LF	OV	EL	ML	LL
Half squat (kg)	88.14 ± 13.7	94.43 ± 14.7	92.63 ± 14.4	89.94 ± 14.0	89.03 ± 13.9	87.24 ± 13.5
Handgrip (kg)	33.83 ± 3.2	32.04 ± 3.2	32.09 ± 3.6	33.31 ± 3.9	32.53 ± 3.3	36.82 ± 4.3
RPE	15.00 ± 1.3	14.61 ± 2.6	14.22 ± 2.0	15.00 ± 1.4	15.17 ± 1.8	15.00 ± 1.8
Motivation (VAS)	7.18 ± 0.9	7.04 ± 1.3	6.91 ± 1.5	7.46 ± 1.4	6.67 ± 1.2	7.35 ± 1.2
Sleep quality (VAS)	6.14 ± 1.2	6.98 ± 1.1	6.29 ± 1.6	6.52 ± 1.6	6.62 ± 1.2	6.65 ± 1.1
Vigor	19.96 ± 2.0	19.63 ± 2.7	17.58 ± 3.4	18.31 ± 2.8	16.89 ± 3.3	15.91 ± 2.9
Fatigue	8.33 ± 1.8	10.26 ± 2.7	10.07 ± 2.5	11.38 ± 1.9	12.01 ± 2.1	12.34 ± 2.6
Depression	3.40 ± 1.1	3.64 ± 1.3	2.63 ± 1.4	3.12 ± 1.0	3.51 ± 1.2	4.51 ± 1.3
Anger	2.37 ± 0.8	1.93 ± 1.2	1.96 ± 0.9	2.26 ± 1.1	2.79 ± 1.0	2.89 ± 1.0

Note. EF = early follicular phase, LF = late follicular phase, OV = ovulation phase, EL = early luteal phase, ML = mid-luteal phase, LL = late luteal phase.

### Cycle effects on physical performance

**Fig. 1:**
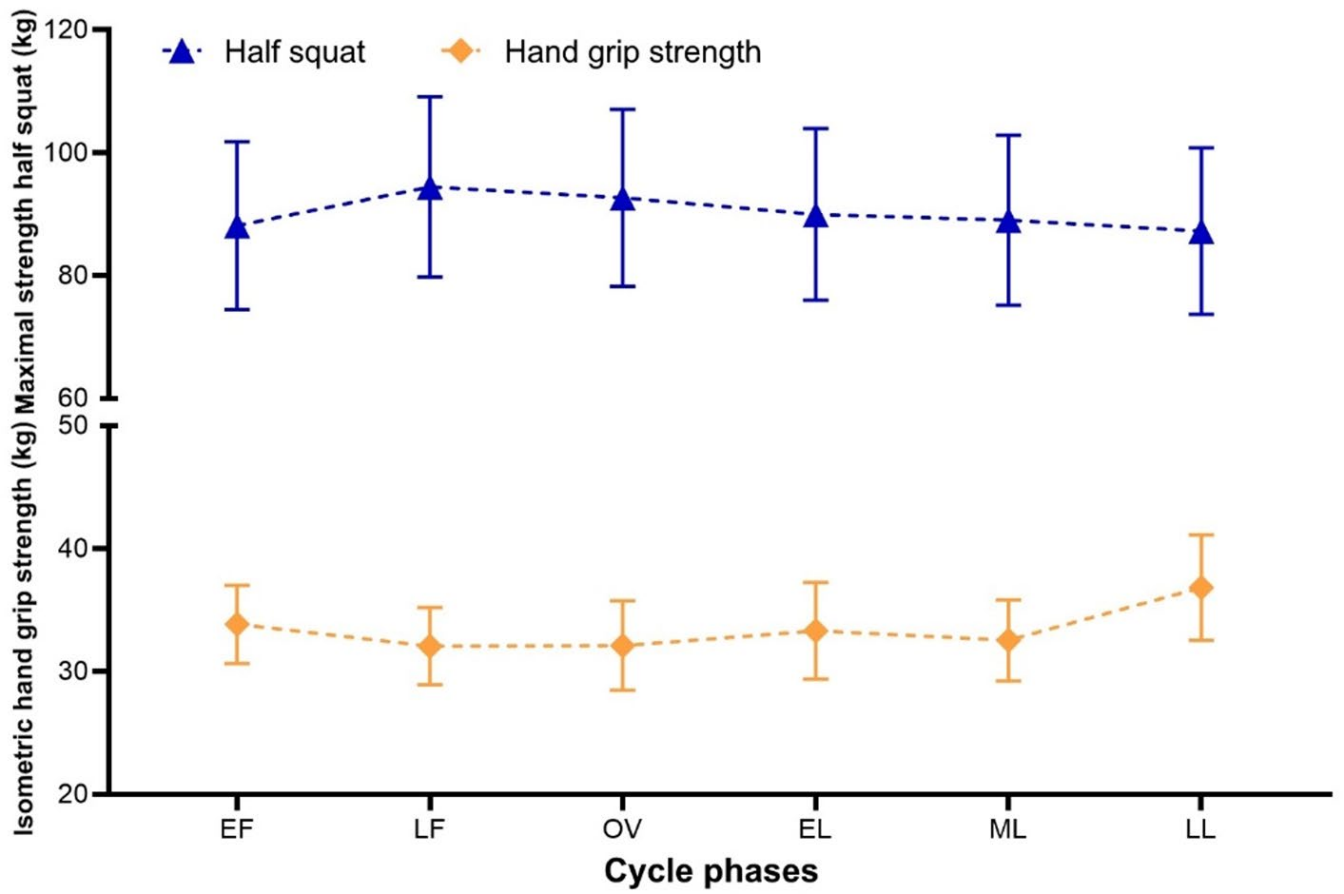
Mean values (M ± SD) of half-squat performance and isometric handgrip strength across six menstrual cycle phases.

Inferential results were consistent with the patterns illustrated in Fig. 1. For isometric handgrip strength, a significant main effect of menstrual cycle phase was observed, *F*(5, 85) = 4.61, *p* <.001, *η*²*p* =.21, with the assumption of sphericity met (Mauchly’s test: *p* =.901). Bonferroni-adjusted post hoc comparisons indicated higher values in the late luteal phase compared with the late follicular phase (*p* =.031), ovulation (*p* =.010), and the mid-luteal phase (*p* =.008). The phase-related increase toward the late luteal phase is reflected in Fig. 1 by the relatively small error bars of the isometric handgrip strength curve.

For dynamic maximal strength in the half squat, a pronounced main effect of menstrual cycle phase was detected. As the assumption of sphericity was violated (*p* <.001; ε(GG) = 0.20), Greenhouse–Geisser–corrected results are reported, *F*(1.01, 17.10) = 737.00, *p* <.001, *η*²*p* =.98. Given the exceptionally large effect size, results should be interpreted with caution, as *η*²p values in repeated-measures designs may be inflated due to low within-subject variability. All pairwise phase comparisons were statistically significant after Bonferroni adjustment (*p* <.001). As illustrated in Fig. 1, mean half-squat values were highest during the late follicular phase and lowest during the late luteal phase, with greater variability compared with isometric handgrip strength. The apparent discrepancy between relatively large standard deviations and the high effect size can be explained by the repeated-measures design, where effect sizes are based on within-subject variability rather than between-subject differences.

Taken together, the results indicate distinct phase-dependent patterns for dynamic and isometric strength measures across the menstrual cycle.

## Cycle effects on motivation and sleep quality

**Fig. 2:**
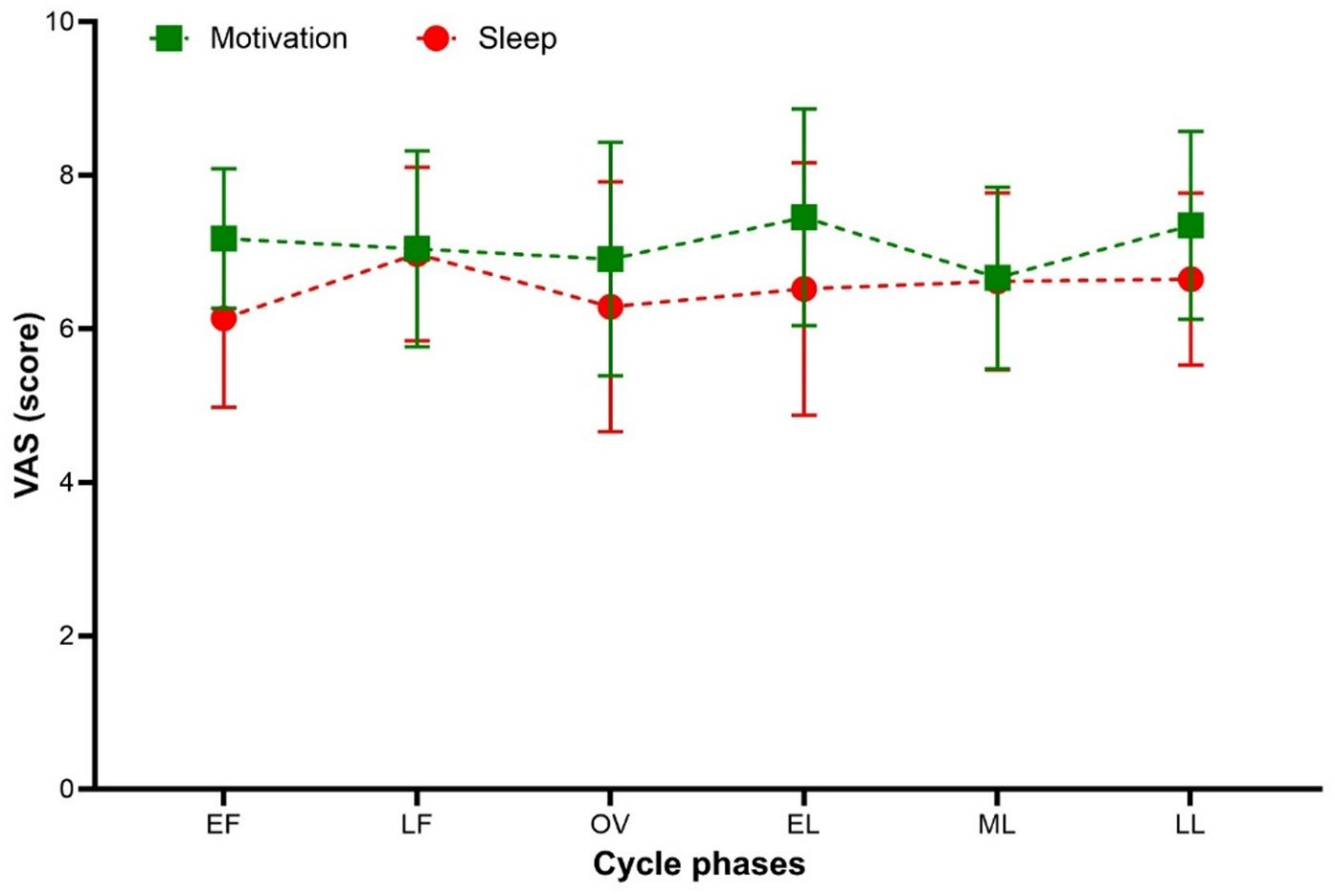
Mean values (M ± SD) of motivation and subjective sleep quality (VAS) across six menstrual cycle phases.

As illustrated in Fig. 2, motivation and subjective sleep quality showed no pronounced variation across the menstrual cycle phases.

For motivation, no significant main effect of menstrual cycle phase was observed, *F*(5, 85) = 0.91, *p* =.479, *η*²*p* =.05. Subjective sleep quality likewise did not vary significantly across phases after Greenhouse–Geisser correction, *F* = 0.97, *p* =.424, *η*²*p* =.05. No significant post hoc differences were detected for either variable.

As shown in Fig. 2, both variables displayed largely stable trajectories across the cycle. Motivation exhibited only minor fluctuations without a distinct peak, while subjective sleep quality appeared slightly lower during the early follicular phase and at ovulation.

### Cycle effects on POMS subscales

**Fig. 3:**
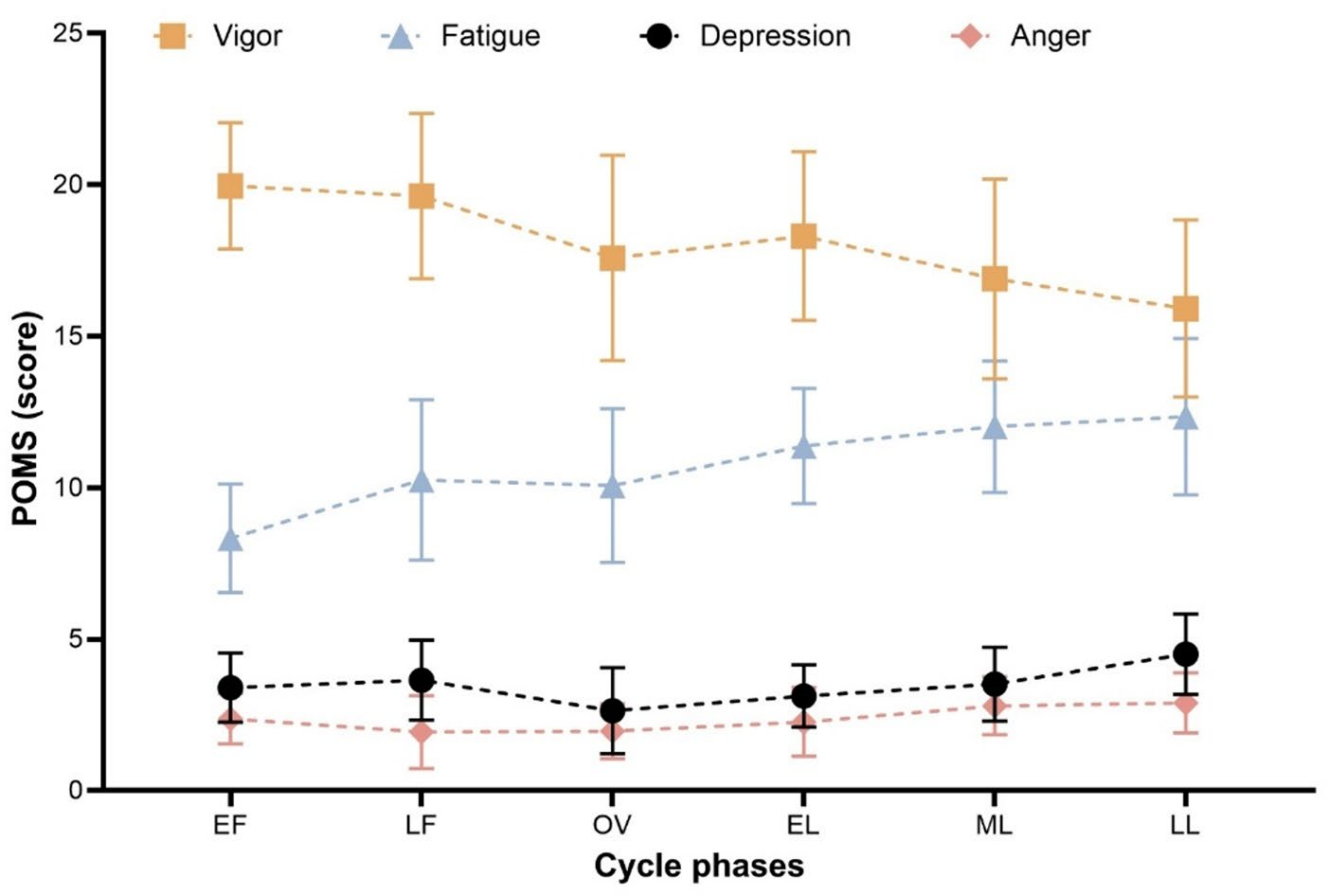
Mean values (M ± SD) of POMS subscales across six menstrual cycle phases.

Phase-related differences were observed across the POMS subscales, as illustrated in Fig. 3. For anger, a significant main effect of menstrual cycle phase was detected, *F*(5, 85) = 2.64, *p* =.029, *η*²*p* =.13; however, none of the Bonferroni-adjusted pairwise comparisons reached statistical significance.

Fatigue demonstrated a clear main effect of cycle phase, *F*(5, 85) = 7.76, *p* <.001, *η*²*p* =.31. Bonferroni-adjusted post hoc analyses revealed higher fatigue scores in the late luteal phase compared with the early follicular phase (*p* <.001), the early luteal phase (*p* =.010), and the mid-luteal phase (*p* =.003).

For vigor, a significant main effect of menstrual cycle phase was observed, *F*(5, 85) = 5.88, *p* <.001, *η*²*p* =.26. Bonferroni-adjusted post hoc analyses indicated higher vigor scores in the early follicular phase (*p* <.001) and late follicular phase (*p* =.025) compared with the late luteal phase.

Depression scores also varied significantly across phases, *F*(5, 85) = 4.63, *p* <.001, *η*²*p* =.21. Post hoc comparisons revealed lower depression scores at ovulation (*p* =.027) and during the early luteal phase (*p* =.041) compared with the late luteal phase.

As illustrated in Fig. 3, vigor decreased toward the end of the cycle, whereas fatigue increased across phases. Depression scores were lower during the early and mid-cycle phases compared with the late luteal phase.

### Associations between physical and psychological variables

**Fig. 4:**
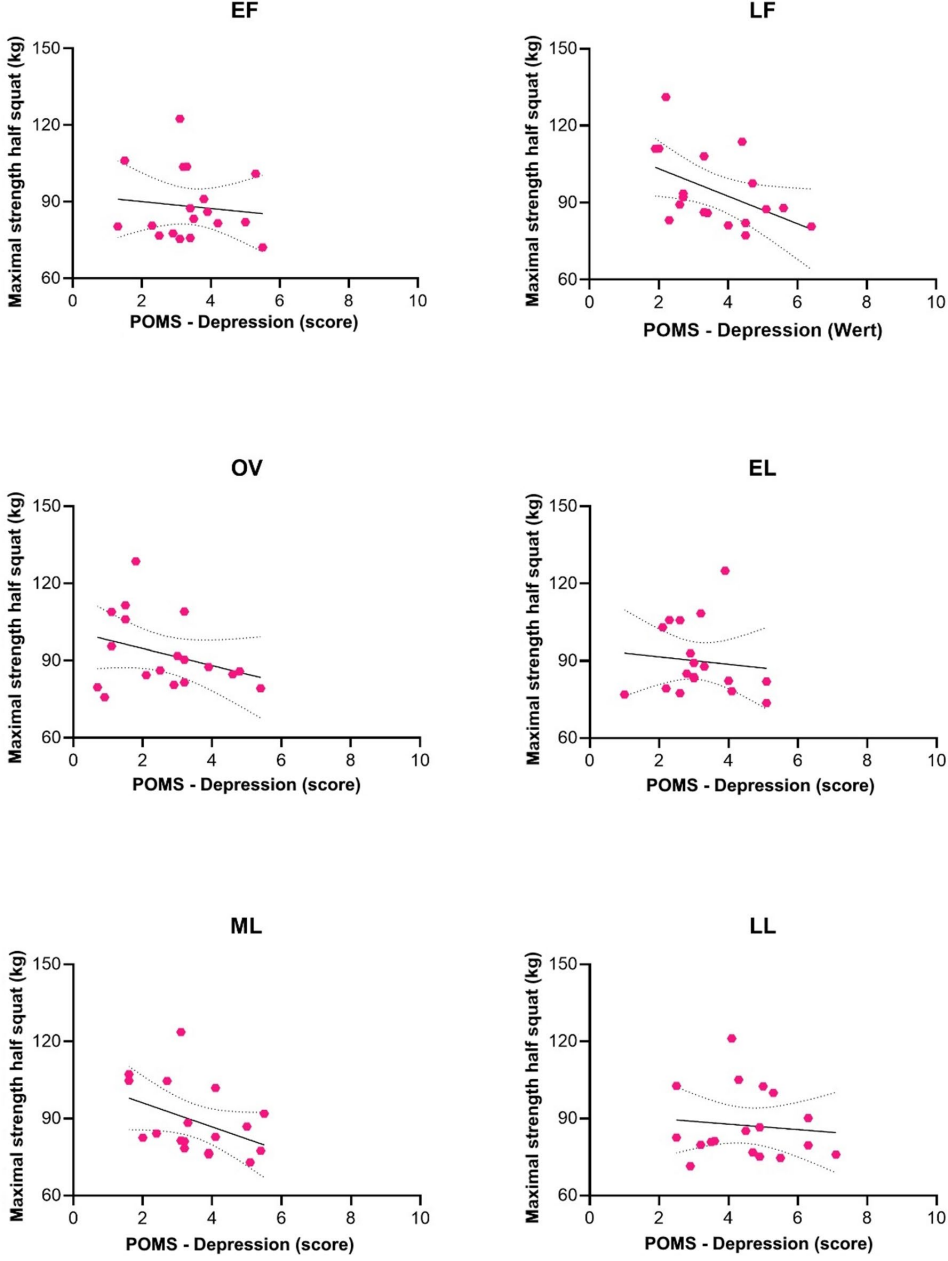
Association between half-squat performance (kg) and POMS depression scores.

Correlation analyses were based on values averaged across the six menstrual cycle phases for each participant. A significant negative association was observed between mean half-squat performance and depression scores (*r* = −.60, *p* =.009). Given the small sample size, this association should be interpreted with caution and does not imply a causal relationship. Figure 4 illustrates this relationship across phases. In the late follicular, ovulatory, and mid-luteal phases, regression lines showed a more pronounced negative slope, whereas associations appeared weaker in the early follicular, early luteal, and late luteal phases.

All other correlations between physical and psychological variables were non-significant. The association between half-squat performance and handgrip strength was weak and positive (*r* =.15, *p* =.554). Correlations between half-squat performance and vigor (*r* = −.12, *p* =.646), fatigue (*r* =.07, *p* =.799), and motivation (*r* = −.24, *p* =.341) were not statistically significant. Likewise, no significant associations were found between isometric handgrip strength and vigor (*r* = −.05, *p* =.849), fatigue (*r* = −.12, *p* =.643), or motivation (*r* =.15, *p* =.556).

Several associations approached but did not reach statistical significance, including perceived exertion and depression (*r* =.47, *p* =.052), motivation and fatigue (*r* = −.43, *p* =.073), and sleep quality and anger (*r* = −.44, *p* =.065).

## Discussion

### Interpretation of cycle effects

The present findings demonstrate phase-dependent differences in both physical performance and psychological parameters across the menstrual cycle. The most pronounced variations were observed in the two strength measures and in the mood-related POMS dimensions. These results support the assumption that hormonal fluctuations across the menstrual cycle can exert measurable effects on physical performance and psychological well-being^1,9^.

Half-squat performance peaked during the early and late follicular phases and around ovulation, whereas the lowest values were observed during the late luteal phase. This pattern aligns with findings by Romero-Moraleda et al.^9^, and the recent meta-analysis by Niering et al.^6^, both of which reported enhanced dynamic strength performance during phases characterized by low to moderate hormonal concentrations. Niering et al.^6^, specifically identified the late follicular phase as favorable for dynamic maximal strength, which corresponds closely to the present trajectory. A potential explanation may lie in the interaction between elevated estrogen and relatively low progesterone concentrations, which has been associated with phase-dependent modulations in neuromuscular function and central nervous system excitability. Experimental evidence indicates that estrogen exerts predominantly excitatory effects on neural activity, whereas progesterone has inhibitory properties, influencing corticospinal excitability and intracortical inhibition^31^. Consistent with this, studies have reported increased voluntary activation and neuromuscular performance during phases characterized by high estrogen and low progesterone concentrations, particularly around ovulation^32,33^. These neuroendocrine mechanisms may contribute to the enhanced dynamic strength observed during the late follicular phase in the present study. In contrast, higher progesterone levels during the luteal phase have been discussed as potentially attenuating neuromuscular activation and energy availability^10^.

The extremely large effect size observed for half-squat performance should be interpreted with caution, as such magnitudes are uncommon in human performance research and may partly reflect the repeated-measures design and low within-subject variability rather than a purely physiological effect. In the present study, greater variability and more pronounced phase-related changes were observed for dynamic strength compared with isometric strength. This may, in part, be influenced by differences in sport-specific training demands within the sample. Strength- and power-oriented training is associated with increases in maximal force production and neuromuscular adaptations, whereas endurance training primarily induces adaptations related to metabolic efficiency and fatigue resistance^34^. Consequently, athletes from strength- and power-oriented sports typically demonstrate higher maximal strength and rate of force development compared with endurance-trained athletes^35,36^, which may have contributed to the observed variability in dynamic strength outcomes.

However, not all studies report consistent cycle-specific strength differences. Colenso-Semple et al.^4^, and Meignié et al.^5^, found no stable phase-dependent effects and attributed heterogeneity primarily to methodological limitations, small sample sizes, and inconsistent phase verification. This underscores the importance of rigorous hormonal validation. By using LH-based phase confirmation, the present study contributes methodologically robust data to an area characterized by substantial variability.

Psychological parameters likewise exhibited phase-related variation. Fatigue increased toward the late luteal phase, whereas vigor and depression scores indicated comparatively more favorable mood states during earlier phases. These findings are consistent with the reviews by Safari et al.^16^, and Gordon et al.^15^, which describe cyclical changes in mood and energy levels and relate them to estrogen-mediated modulation of serotonergic and dopaminergic systems. The elevated fatigue observed in the late luteal phase aligns with studies reporting increased neuromuscular fatigue and subjective tiredness during this phase^7^. In contrast, sleep quality remained relatively stable, consistent with findings by Alzueta and Baker^13^, who reported subjective fluctuations without consistent objective alterations. The integration of endocrine stress markers, as discussed by Cook et al.^8^, provides an additional framework for understanding phase-dependent variations in readiness and performance.

The absence of significant phase effects for motivation and perceived exertion suggests that not all subjective states are equally sensitive to hormonal fluctuations. It is conceivable that trained athletes compensate for cyclical variations through adaptive training behaviors or established self-regulatory strategies. This interpretation is in line with Gordon et al.^15^, who highlight the buffering role of psychological regulation and habitual routines in mitigating hormonally driven performance variability.

Overall, the findings indicate that menstrual cycle–related hormonal fluctuations are associated with measurable, yet variable, effects on both physical and psychological parameters. The direction of effects largely corresponds with current literature, while variability in magnitude and expression underscores substantial interindividual differences^1,10,12^.

### Interpretation of associations between physical and psychological variables

The second part of the research question addressed the relationship between physical performance and psychological well-being. A significant negative association was observed between mean half-squat performance and depression scores, indicating that athletes with higher maximal strength tended to report lower levels of depressive mood. This finding aligns with broader sport psychology literature demonstrating mood-stabilizing and stress-reducing effects of regular physical activity^2,15^. Similarly, Alzueta and Baker^13^, reported positive associations between perceived energy and physical activity levels across the menstrual cycle.

However, evidence in this domain remains inconsistent. Meignié et al.^5^, reported no stable correlations between physical and psychological parameters and attributed this heterogeneity to differential hormonal pathways. While estrogen may enhance neuromuscular activation, progesterone appears to exert stronger effects on affective and cognitive regulation. Colenso-Semple et al.^4^, likewise emphasize that interactions among hormones, mood, and performance are unlikely to follow linear patterns and may be moderated by individual sensitivity and contextual factors.

The absence of significant associations between motivation, sleep quality, and physical performance further suggests that these constructs may operate relatively independently within trained athletes. It is plausible that external influences, such as training environment, social support, or daily stressors, exert stronger effects on these variables than cyclical hormonal fluctuations. In addition, isometric handgrip strength has been described as less sensitive to hormonal variation compared with dynamic multi-joint performance tasks^12^, which may partly explain the lack of consistent associations.

Differences between subjective and objective markers may also be influenced by energy availability. Grabia et al.^37^, highlight that energetic imbalances can simultaneously affect menstrual function, mood, and performance, suggesting that endocrine and metabolic factors should be considered when interpreting individual trajectories.

Overall, the findings indicate that while hormonal influences on physical and psychological variables are detectable, their interrelationship appears complex and likely mediated by multiple interacting mechanisms. The interplay between menstrual cycle phase, performance, and mood should therefore be understood as a multifactorial process requiring integrative consideration of endocrine, physiological, and psychosocial determinants^1,38^.

### Limitations

Despite the systematic design and hormone-based phase validation, several limitations should be considered when interpreting the findings. The sample comprised 18 athletes, and although powered to detect medium-sized effects, smaller effects cannot be ruled out. Furthermore, while the relatively homogeneous sample enhanced internal comparability, generalizability to other age groups, performance levels, sports, women using hormonal contraception, or individuals with irregular menstrual cycles is limited.

In addition, the inclusion of athletes from different sport disciplines may have introduced variability in strength performance due to differences in training background and sport-specific demands, which should be considered when interpreting the results.

Cycle phase classification was based on urinary LH testing, which provides greater accuracy than calendar-based methods. However, this approach does not replace comprehensive hormonal profiling. Thus, precise hormonal exposure within each phase cannot be fully determined. Individual variation in cycle length and the possibility of anovulatory cycles also cannot be entirely excluded. The use of urinary LH testing was chosen as a non-invasive and practically feasible approach for repeated measurements in this study.

Training load, nutritional intake, sleep behavior, and psychosocial factors were not experimentally controlled between testing sessions. These variables may influence both performance and mood states and could attenuate or amplify phase-related effects. In addition, several psychological measures were based on self-report, which may be susceptible to day-to-day variability and expectancy bias.

### Practical implications

The present findings indicate that both physical performance and psychological well-being exhibit measurable variation across the menstrual cycle in physically active women. Higher strength and vigor values were observed during earlier cycle phases, whereas increased fatigue and depressive mood were evident toward the late luteal phase. In addition, a negative association was identified between maximal strength and depressive symptoms, suggesting a potential interaction between physical and affective dimensions.

These results have implications for applied sport settings. While phase-related variations were detectable at the group level, interindividual variability was substantial. Consequently, rigid phase-based training prescriptions may be less appropriate than individualized load management approaches. Monitoring both physical and psychological markers may provide a more nuanced understanding of performance readiness across the cycle.

From a practical perspective, structured but flexible monitoring strategies may be beneficial. Regular assessment of subjective indicators such as fatigue, mood, and perceived recovery could support informed decision-making in training and rehabilitation contexts. Integrating simple self-report measures alongside objective performance data may help identify individual patterns and improve responsiveness to fluctuating readiness states. However, individual responses should take precedence over phase-based expectations, and training decisions should primarily be guided by the athlete’s current subjective and objective readiness.

Importantly, cycle-sensitive training does not necessarily require complex structural adaptations. Rather, informed communication, awareness of cyclical patterns, and individualized interpretation of performance and well-being data may enhance athlete-centered coaching practices. Future research should evaluate whether structured cycle-informed feedback systems improve training quality, adaptation, and psychological well-being in athletic populations^17^.

## Data Availability

The original contributions presented in the study are included in the article/supplementary material, further inquiries can be directed to the corresponding author/s.
